# A survey on server-based electronic identification and signature schemes to improve eIDAS: with a new proposal for Turkey

**DOI:** 10.7717/peerj-cs.734

**Published:** 2021-10-13

**Authors:** Ozgun Erdogan, Nurdan Ayse Saran

**Affiliations:** 1 Innova Information Technology Solutions, Ankara, Turkey; 2 Department of Computer Engineering, Cankaya University, Ankara, Turkey

**Keywords:** Server-based e-ID, Mobile signature, Server-based e-signatures, Turkish e-ID, eIDAS

## Abstract

The design, development, and implementation of e-Government applications aim to improve the quality of daily life and facilitate the mobility of citizens by reducing the constraints imposed by existing borders. This study examines previous research in the literature on electronic identification (eID) credentials technologies and the projects carried out in Europe. This study focuses especially on server-based e-signing methods. In the light of these reviews, the applicability of a server-based mobile electronic signature model without disrupting local initiatives has been examined as a case study. As an exemplary case, Turkey’s eID structure is examined from a technical and legal perspective. When creating the proposed server-based eID model, it was especially inspired by Austria’s server-based approach in use. In this process, the suitability of the existing structure with the server-based e-signing method was examined. In addition, some suggestions were made to eliminate the problems that may prevent the use of the proposed server-based e-signing method. This study revealed that a server-based electronic signature approach would develop a more user-friendly and flexible solution in identity management. It was concluded that using a server-based signature approach would help achieve international standards for cross-border online identification methods.

## Introduction

Electronic identification (eID) is gaining more and more importance as our lives shift to today’s digital realm. For a successful and efficient e-Government system, countries develop services so that citizens can process their requests without physically visiting public offices. It is essential in the globalizing world that electronic identity applications can be used in one country and between countries. Therefore, governments need to develop ways to integrate digital services. Significant progress has been made in this regard in Europe. Since 1999, digital services of EU member states have been developed on the basis of a common legal framework, Directive 1999/93/EC (https://eur-lex.europa.eu/legal-content/EN/TXT/?uri=celex%3A31999L0093, retrieved 24.05.2021). The current eIDAS Regulation (https://ec.europa.eu/futurium/en/content/eidas-regulation-regulation-eu-ndeg9102014.html), is a continuation of the old directive representing “electronic Identification, Authentication and Trust Services”. This Directive regulates electronic identity and trust services for electronic transactions to develop the single digital market in the EU. Under eIDAS, citizens and businesses can use their local eID when accessing public services in the other EU Member States using electronic IDs. The Regulation also sets standards for electronic signatures, timestamps, electronic seals, and additional authentication proofs. In this way, cross-border mutual recognition of electronic identity within the European Union is ensured.

Qualified electronic signatures are required for safe and trouble-free electronic transactions between countries. This study examines identity registration and verification techniques, which will be the primary layer of e-government architectures that can work across countries. This review aims to identify the main issues and problems that should be considered while developing an internationally operable e-identity system. Today, an authentication approach based on smart cards distributed by certificate authorities is widely used in e-identity systems. However, this approach has many disadvantages, both in terms of usability and cost (see in “Usability”). In the literature, a server-based signature scheme, an alternative approach that eliminates the existing disadvantages and enables international interoperability, has been proposed (Orthacker, Centner & Kittl, 2010). In addition, server-based schemes can increase usability by eliminating the need for end-users to carry smart cards and the card’s loss or theft. It can also support the collection of homogeneous data by avoiding the use of different typologies for the Member States at the European level, thereby increasing cross-border interoperability. In addition, essential security requirements have been established for all components and services to create qualified electronic signatures with eIDAS. Server-side solutions can address these requirements in server-based remote signing methodolgy, and security concerns can be avoided. One of the primary aims of this study is to demonstrate how an alternative server-based signature scheme can be used for eID environments without disrupting local initiatives (Hühnlein et al., 2019). In this process, as a case study, we examined how a server-based e-signature method can be integrated into existing e-signature applications in Turkey, whether it will facilitate e-signature processes, and its compatibility with existing systems. In this case study, we identify the necessary steps to integrate a server-based e-signature method into the existing infrastructure successfully. The results of this study can significantly increase the applications of server-based electronic signature schemes in different sectors (Aciobanitei, Luculescu & Pura, 2020; Aciobanitei et al., 2020), thus accelerating the realization of the technology-neutral eIDAS vision.

### Survey methodology

In this study, we first present the current developments in eID technologies to reveal the barriers and technical difficulties that may arise in adapting eID technologies to national government systems using a deductive research approach (Yin, 2009). We then focus on server-based remote signing technology and present a general theoretical framework for the applicability of this technology through a case study.

When examining eID systems, social, economic, political, technical, and technological factors should be considered. However, the focus of attention in this study is technical and technological factors.

The World Bank^1^ 1Technology Landscape for Digital Identification, https://documents1.worldbank.org/curated/en/199411519691370495/Technology-Landscape-for-Digital-Identification.pdf, retrieved 23/05/2021. has grouped technologies used for identification and authentication into three broad categories: credentials (*e.g*., cards, mobiles), authentication and trust frameworks, and analytics (risk assessments). Risk assessment is out of the scope of this paper.

Research Questions are as follows:

- What are the advantages and disadvantages of the current widely used Secure Signature Creation Devices (SSCDs)?
- Is it possible to reduce digital fragmentation and increase cross-border interoperability in the use of e-services by using remote signing with mobile solutions?
- Can remote signed eIDAS be implemented in Turkey without breaking the existing structure of local initiatives?

### Search process

The study aims to review relevant research and the body of knowledge available to provide a holistic approach to research questions. All technical terms have been conveniently introduced and explained. While searching for relevant sources, a desk study (previous studies, reports, regulations, EU Commission’s web pages, articles, etc.) has been conducted. In order to carry out this kind of research, we examined our search results in two dimensions; regulations and academic studies. The keywords “e-ID” and “e-IDAS regulation” for both “in Europe” and “in Turkey” has been searched in Google Scholar resulting in 177 articles. Also, the keywords “identity management’’ and “server-based signature” have been searched in Google Scholar resulting in 11 articles. The reviewed papers are mainly in English. The subject area covered is computer sciences. We excluded the articles that focus on cryptographic implementation techniques such as blockchain, pairing-based, and specific applications for universities, medical, financial. Also, we excluded the papers that focus on legislation, law, strategy, and justice. For the regulations, the search strings created to retrieve information from the site: ec.Europa.eu, site: www.resmigazete.gov.tr (for Turkish regulations) and also The Information and Communication Technologies Authority (ICTA) (Turkish: Bilgi Teknolojileri ve İletişim Kurumu (BTK)), General Directorate of Population and Citizenship Affairs (Turkish: Nüfus ve Vatandaşlık İşleri Genel Müdürlüğü). Since we are focusing on the European eID system, we excluded the projects and regulations outside the European Union.

This study is intended for the benefit of government policymakers, other relevant public officials, as well as private sector partners and researchers. This article’s primary purpose is to present the components of a common electronic identity approach regarding eIDAS regulations and reveal the current situation, especially in the Member states.

The article’s organization is as follows: “Background” primarily discusses the main concerns of developing e-ID technologies. Next, we define the authentication factors, give the advantages/disadvantages of Secure Signature Generating Devices (SSCD) as a possession factor, and the features that a qualified electronic signature should provide. We also provide the security assurance level, technology, and standards that an SSCD must meet. In “E-ID in Europe” and “E-ID in Turkey”, we provide an overview of current relevant studies in Europe and present the current situation in Turkey. The proposed study reviews 20 papers that summarize country cases and four projects in Europe to answer the research questions. The following section presents the remote signing e-Identity System framework and the technical details required to implement it. Next, “Discussion” discusses the applicability of the method. Finally, “Conclusions” concludes the study.

## Background

This section focuses on the basic concepts of electronic identity, which form the basis of the study. First of all, the basic issues related to eID will be mentioned, then authentication factors, secure signature creation devices, qualified electronic signature, security assurance levels, and eID technologies and standards will be discussed, respectively.

### Main issues in eID

There are many important issues related to electronic identification. Beyond privacy concerns, interoperability and usability must also be met by electronic identification in eIDAS. This section explains these important concepts.

#### Privacy

The Government-issued/recognized eID of a person contains mainly three parts;

- identification data such as a photo or biometric data
- possible user profile data such as citizenship
- authentication credentials such as digital certificate and the corresponding private key (for authentication and digital signing purposes)

The collection and processing of Personally Identifiable Information (PII) may bring privacy concerns (Szadeczky, 2018). Hence, it is necessary to associate the claimed identity with the applicant providing identity evidence for appropriate identity validation and verification. Therefore, it should be limited to the minimum required to validate the existence of the claimed identity. Also, it should prevent unauthorized access to the identity token (Dumortier & Vandezande, 2012; Han, Chai & Liu, 2012).

#### Usability

Usability of electronic identification is a challenging task since eIDAS promotes the seamless and widespread use of secure eID across the Member States. According to commission report^2^ 2The user experience of eIDAS-based eID, https://ec.europa.eu/cefdigital/wiki/download/attachments/52600425/20190206_eID_Main_report.pdf?version=1&modificationDate=1551177429365&api=v2, retrieved 24.5.2021., it should be useful, usable (easy to use), desirable (images and other design elements should be used to evoke emotion and appreciation), findable (content needs to be navigable and locatable onsite and offsite), credible (users must trust and believe what you tell them) (Zefferer et al., 2014).

#### Interoperability

It is defined as “the ability of a system or a product to work with other systems or products without special effort on the part of the user, covering both the holder of the eID and the counterparty on the receiving end of electronic communication” (Myhr, 2008; Mocanu et al., 2019). Two main issues may be listed as cross-border operability and fragmentation.

- Cross Border Operability: A pan-European eID is closely connected with cross-border operability. It is defined as “a citizen from one country to have access to an application in another country” (Leenes et al., 2009). Due to the increasing mobility of citizens, citizen identification across borders is a major issue that needs a solution for maximizing the potential of cross-border service. Therefore, interoperability across the border of national electronic identity management systems (Melin, Axelsson & Söderström, 2016) becomes more and more important (Sideridis et al., 2017; Sedek, Sulaiman & Omar, 2011).
- Fragmentation: Fragmentation is also a common problem with identification systems. It separates identification structures to satisfy sector-specific demands without establishing standards (Rivera, Milena & Kristjan, 2015). The same basic information is collected from the users repeatedly and used for authentication purposes in separate databases of institutions such as government institutions, hospitals, telecommunication companies, and banks. As explained in the FORMIT (2013) Foundation’s e-Signature Final Study Report: “Existence of different typologies of e-signature has allowed the Member States to apply the Directive (https://eur-lex.europa.eu/legal-content/EN/TXT/?uri=celex%3A31999L0093) with certain degrees of freedom, generating more confusion than opportunities in cases that require interoperability across the national boundaries.” Adding different implementations and sector-based solutions to provide identity validation alternatives causes the overall system’s usability to get more complicated for the end-users.

Also, accountability, and transparency are fundamental issues in identity management systems (Protopappas, Sideridis & Yialouris, 2020).

### Authentication factors

Authentication factors can be divided into three categories (SecSign (2019)):

- Knowledge-based factors (what-you-know)
- Possession-based factors (what-you-have)
- Inherent factors (what-you-are)

The authentication system may be either two-factor or three-factor depending on the number of parameters (Patel et al., 2020), and multi-server systems are preferable in eID technologies. At least two different authentication factors are mandatory for such a system (Nikolouzou, 2020). Biometrics are used as inherent factors. In recent years, multi-modal and behavioral biometrics have become more popular in terms of security. Since mobile devices can verify transactions using built-in sensors, multi-modal biometric authentication is also preferred. Various studies investigate the reliability and security of biometrics. The different factors should be chosen to counter different threats/attack vectors. But in this study, we focused on possession and knowledge, ignoring the third factor for simplicity.

### Secure signature creation devices (SSCD)

A secure signature creation device (the synonymous “QSCD” under eIDAS) is software or hardware that has been configured to generate an electronic signature as defined in eIDAS. Along with the developments in two-factor authentication schemes, different e-ID credentials technologies have been proposed, such as identification *via* smart card methods, mobile methods, citizen card concepts, etc. These solutions’ advantages and disadvantages are listed below.

- Smart Card Solutions: There are various types of smart cards; contact/contactless smart cards. They connect to a reader either by physical contact or remote short-range interfaces such as radio-frequency identification (RFID) or near-field communication (NFC). USB tokens can also be examined similar to smart cards.
  - **Advantages:** stability, low level of usability (FORMIT, 2013). Since being the first e-signature solution, it became a regular practice in time; people could not abandon their habit easily.
  - **Disadvantages:** the necessity for specialized data input devices in combination with the associated software and location dependency (Rath et al., 2014); lack of necessary infrastructure and applications, high cost of transition, and integration to electronic environments, low awareness/lack of awareness (Kabasakal, 2007). Another consideration is the risk of losing the token. Such solutions should technically assure that the data cannot be read from the card’s chip.
  - Citizen Card Solutions
    –**Advantages:** adding more features such as e-signatures, containing fingerprint and biometric information of one (Stipsits & Kammerstetter, 2015).
    –**Disadvantages:** hardware dependency (using a national ID card from one EU Member State to another Member State is not possible yet (Huhnlein et al., 2016)).

European Committee for Standardization has an architecture for a European interoperable eID system within a Smart Card infrastructure, (https://joinup.ec.europa.eu/collection/european-committee-standardization-cen/solution/architecture-european-interoperable-eid-system-within-smart-card-infrastructure/about), retrived 24.05.2021. Notified schemes based on eID cards with LoA (See in Security Assurance) high in 2019 (Italy, Estonia, Spain, Croatia, Luxembourg, Belgium, Czech Republic, Latvia, Lithuania, Slovakia, Germany, Portugal)^3^ 3Overview of Pre-Notified and Notified eID schemes under eIDAS, https://ec.europa.eu/cefdigital/wiki/display/EIDCOMMUNITY/Overview+of+pre-notified+and+notified+eID+schemes+under+eIDAS, retrieved 24.05.2021..

- Mobile Solutions: Mobile technologies have various international standards for secure crypto applications such as OTP (One Time Pad), Mobile Connect (an initiative from the GSMA that aims to provide new digital authentication standards)^4^ 4Mobile Connect, https://www.gsma.com/identity/mobile-connect, retrieved 24.05.2021.. Besides these, trusted hardware devices enable secure storage and isolated processing of sensitive data. Such as TPM (Trusted Platform Module by the Trusted Computing Group), ARM TrustZone, Trusted Execution Environment (TEE), and electronic chips called Secure Elements (*e.g*., UICC (Universal Integrated Circuit Card), also known as Cryptographic SIM, NFC secure elements) (Nyman, Ekberg & Asokan, 2014).
  –**Advantages:** ease of use; low cost to the user, and the possibility of signing independent of time and space.
  –**Disadvantages:** the dependence on specific use cases (Rath et al., 2014), security (according to Ruiz-Martnez et al. (2007)) “mobile handsets” have reached a significant penetration rate in many countries such as Luxembourg (164%), Italy (128%), Hong Kong (117%), Spain (109%), Chile (74%), Argentina (64%), and so on. Also, the risk of malware exposure is a problem since cryptographic operations, and private data are contained in SIM cards (Rath et al., 2014).

Countries Austria, Belgium, Estonia, Finland, Germany, Iceland, Latvia, Lithuania, Norway, and Sweden, have completed their studies and launched their mobile eIDs (Sagıroglu, Kabasal & Alkan, 2008). According to Leitold & Konrad (2019), mobile signature exceeds smart-cards by far. Increasingly, mobile phones are a platform for fingerprint, voice, and facial recognition. Therefore, using mobile phones in place of dedicated hardware may be a more affordable way to bring biometric authentication.

- Server-Based Solutions: In contrast to other solutions, in server-based solutions, the SSCDs are not under the physical control of the user. It is centrally implemented and shared among all users.
  –**Advantages:** eliminates hardware dependency (since no reader or additional software is required from the end-users side), cost-effective for large-scale deployments, user-friendly, and flexible solution (Orthacker, Centner & Kittl, 2010). There is no need for dedicated signing hardware on the user side. Also, it may be seamlessly integrated with mobile devices, web browsers, or client-side applications.
  –**Disadvantages:** most of the server-based approaches are tailored to specific use cases (Rath et al., 2014). User and server authentication are required. Most of them use SMS functionality while signatory proves his/her identity to the server, but not bound to SIM card or mobile network operator.

The usage of HSM (hardware security module) devices increases the security level (Orthacker, Centner & Kittl, 2010). It offers central management of eID services with proper standards, the integration of these separate services is provided at a sufficient level (Rath et al., 2014).

### Qualified electronic signatures (QES)

With the introduction of Directive 1999/93/EC (https://eur-lex.europa.eu/legal-content/EN/TXT/?uri=celex\%3A31999L0093) that is Community Framework for Electronic Signatures prepared by the European Commission, e-signatures that meet certain conditions became legally equivalent to the wet signatures. Therefore, QES needs to fulfill several requirements and be created in an SSCD based on a qualified certificate. According to Directive, an advanced electronic signature is a signature that is

- uniquely linked to the signatory
- capable of identifying the signatory
- created using means that the signatory can maintain under his sole control
- linked to the data to which it relates in such a manner that any subsequent change of the data is detectable

According to Directive, a qualified electronic signature is a signature that is

- based on a qualified certificate (specified in Annex I of the Directive)
- created by a secure signature creation device that needs to comply with Qualified Certificates requirement.

In practice, the underlying technology is based on public key infrastructure (PKI). We want to draw reader’s attention that according to eIDAS, QSCD (Qualified Signature Creation Device) has to be operated by a QTSP (Qualified Trust Service Provider)^5^ 5Conformity Assessment of Qualified Trust Service Providers, https://www.enisa.europa.eu/publications/assessment-of-qualified-trust-service-providers..

### Security assurance levels

While assessing an authentication mechanism, the threats that should be taken into account are online guessing, offline guessing, credential duplication, phishing, eavesdropping, replay attacks, session hijacking, man-in-the-middle, credential thefts, spoofing, and masquerading in the ISO 29115^6^ 6ISO/IEC 29115:2013–Entity authentication assurance framework for additional information, Section 2.1.2.2..

The STORK^7^ 7STORK, pan-European electronic-identity authentication project, https://ec.europa.eu/digital-single-market/en/content/stork-take-your-e-identity-you-everywhere-eu. project has created four Quality Authentication Assurance (QAA) levels. Level 4 is the most reliable, and level 1 is the least reliable. Currently, three levels of assurance (LoA) are accepted as high, substantial, and low, based on ISO 29115, STORK [STORK-D2.3], NIST [NIST-800-63-2] and CIR 2015/1502^8^ 8Commission implementing Regulation (EU) 2015/1502 of 8 September 2015 on the minimum technical specifications and procedures for assurance levels for electronic identification..

### Technologies and standards in eID

There are many technologies and standards related to eID. In this part, first, the most widely used technologies are listed. Then, the most widely used standards and frameworks are presented.

- For secure information exchange-SAML (Security Assertion Markup Language-single sign on communication)
- For endpoint security-TLS (Transport Layer Security—a cryptographic protocol that provides communication security over a computer network)
- For identity authentication systems on mobile devices and web applications-FIDO UAF (Fast Identity Online Universal Authentication Factor), FIDO U2F (Universal 2nd Factor)^9^ 9Note that FIDO U2F is developed in FIDO2 project; WebAuthn (Web Authentication; on device authenticator, web authentication and through browser) and CTAP(Client to Authenticator Protocol) are the primary outputs; CTAP is a user-controlled cryptographic authenticator (such as a smartphone or a hardware security key).
- For delegating access-oAuth
- For extra identity layer built on top of OAuth-OpenID connect
- For advanced electronic signatures formats^10^ 10Note that although CEF (Connecting Europe Facility) supports the ETSI EN 319 xxx standards released in 2016, please note that Decision 2015/1506/EU still refers to the previous set of standards.,-XAdES^11^ 11XML Advanced Electronic Signatures (XAdES) Baseline Profile-ETSI, https://ec.europa.eu/cefdigital/wiki/display/CEFDIGITAL/eSignature+standards#eSignaturestandards-XAdES(XMLAdvancedElectronicSignatures)BaselineProfile., CAdES^12^ 12CMS Advanced Electronic Signatures Baseline Profile, https://ec.europa.eu/cefdigital/wiki/display/CEFDIGITAL/Standards+and+specifications., PAdES^13^ 13PDF Advanced Electronic Signature Baseline Profile, https://ec.europa.eu/cefdigital/wiki/display/CEFDIGITAL/Standards+and+specifications. or ASiC ETSI Plugtests Baseline Profile^14^ 14Associated Signature Container Baseline Profile, https://ec.europa.eu/cefdigital/wiki/display/CEFDIGITAL/Standards+and+specifications..

Standards and Frameworks: Standardization is a critical issue to achieve the reduction of dependence on external suppliers, to prevent abuse of dominant market positions, to assure transparency, security and interoperability (Blind & Gauch, 2009; Werle & Iversen, 2006; Sealed & Piper, 2007). The whole list of standards for eIDs and TSPs are listed in Enisa Report ’’Standardisation in the field of Electronic Identities and Trust Service Providers’’^15^ 15Standardisation in the field of Electronic Identities and Trust Service Providers, https://www.enisa.europa.eu/publications/standards-eidas.. The most widely used standards and frameworks are listed below:

- ISO/IEC 29003:2018—Identity proofing
- ISO/IEC 29115:2013—Entity authentication assurance framework
- NIST SP 800-63—Digital Identity Guidelines
- CEN/TR 419010—Framework for standardization of signature
- PKI Standards-X.509 (ITU) for the certificate format, PKIX (IETF) standards for core PKI and PKCS standards for interfacing to secure devices
- CEN 419241-Trustworthy Systems Supporting Server Signing.

## E-ID in Europe

In the beginning, countries formed their country-based solutions in order to get the most benefit from electronic signatures. After 2007, pilot projects like STORK1.5 (Secure Identity Across Borders Linked) (Leitold, 2011), STORK 2.0, and e-SENS^16^ 16e-SENS, http://www.esens.eu/, retrieved 24.05.2021. were started by EU organizations to support the cross-border identity validation (Eichholtzer, 2009). STORK is a Large Scale Pilot project that gathers 58 organizations from 19 Member States (Leitold & Posch, 2012). e-SENS was launched in 2013 gathers 100 organizations from 22 countries. As the eIDAS regulation (https://ec.europa.eu/futurium/en/content/eidas-regulationregulation-eu-ndeg9102014) introduced electronic identity and trust services for international electronic transaction schemes in 2014, researchers once again gave some thought to a global eID concept. A global project named FutureTrust^17^ 17FutureTrust, https://cordis.europa.eu/project/id/700542, retrieved 24.05.2021. continues addressing a more straightforward and international way of online identification scheme (Huhnlein et al., 2016). e-Authentication (e-AU), e-Signature (e-SIGN), and e-Identification (e-ID) are used for handling governmental and administrative official procedures or services. Beyond these global projects, several projects have been conducted in Member States that reference architecture for the remote provision of eIDAS-related services such as SkIDentity^18^ 18SkIDentity, retrieved July 2021, https://www.skidentity.de/. and FutureID^19^ 19FutureID, retrieved July 2021, https://cordis.europa.eu/project/id/318424. (Hühnlein, 2014). Another project, named Eksistenz, deals with security concerns such as ID theft (Liu-Jimenez et al., 2015).

As the technology of e-signatures continues to improve, countries continue to try to adapt their identity structures to the new advanced identity validation methods to achieve a more secure and usable work environment. However, with the different e-signature solutions used in other sectors, sector-based solutions and use cases are formed. According to the FORMIT (2013) Foundation’s report, the member states have announced plans for a more open, accessible, and transparent administration using the latest electronic signature technologies. Throughout implementing the latest technologies, different typologies like server-based e-signature technologies are formed in order to provide a solution for these problems. The advantages and disadvantages of the latest technologies have been given in the preceding section.

Finland was the first country to issue a national eID card in 1999 (Rissanen, 2010). However, many countries have adopted national ID Card based identification and authentication methods over the past decades, including EU member states Belgium, Estonia, Austria, Sweden (Göransson, 2018), Italy, Spain (Gómez de la Cruz, 2005), Portugal, and Germany, etc. (Roßnagel et al., 2012). Estonia used the first national ID card with digital signatures in October 2002 (Rivera, Milena & Kristjan, 2015). Estonia also constitutes an excellent example with its successful integration system that connects the population register records with eID systems (Rivera, Milena & Kristjan, 2015). Although the id card-based method is the direction most countries seem to follow, it is still not possible in practice to use a national ID card from one EU Member State to another Member State just yet (Huhnlein et al., 2016). In addition to the classic national eID card solutions, member states have also notified schemes based on mobile solutions such as Estonian *Mobiil-ID*, the Latvian *eParaksts*, the Portuguese *Chave Móvel Digital*,the Belgian *FAS/itsmee* (Mariën & Van Audenhove, 2010; Soyez, 2019), the Danish *NemID* (Nikolouzou, 2020), the Moldavian (Rosca, 2017). However, it brings a high and increasing mobile penetration in Europe (85% in 2017 and estimated to go up to 88% in 2025) (GSMA, 2018). However, Estonia’s and Austrian e-identity schemes (Stranacher et al., 2013) may be good examples of handling mobility problems (Kubach et al., 2015). In Estonia, the e-residency program developed an integration model ensuring the development of a unified system. The Austrian government first introduced the national card concept then improved their system towards server-based mobile eID strategies supporting cross-border identification of users (Paulin, 2012; Aichholzer & Strauß, 2010; Behrens, 2012; Strauß & Aichholzer, 2010). Austrian and Estonian e-government systems also constitute a base for cross-border identification of citizens. Server-based identity and signature solutions have recently gained popularity in recent years (Rath et al., 2015; Lenz, Stranacher & Zefferer, 2013; Zefferer, 2014; Kubach et al., 2015).

## E-ID in Turkey

During the ongoing process of national citizen cards and studies towards the new e-signature law in 2019, the necessary steps to achieve a more practical and international online identification method are investigated in this study. In order to provide more user-friendly identity validation for Turkish citizens, the private sector and the Turkish government are continuously trying to adapt to the new solutions of identity validation arising from different technological developments. While these new technologies are implemented in the existing system over the years, different typologies of these solutions are formed, sector-based solutions are developed, and general concepts of cross-border operability and usability are overlooked. One of the main reasons for these heterogeneous implementation styles is the lack of clarity in implementation in Directive 1999/93/EC (https://eur-lex.europa.eu/legal-content/EN/TXT/?uri=celex\%3A31999L0093), the community framework for electronic signatures prepared by the European Parliament. In Turkey, different applications using different e-signature technologies results in different passwords for a single user. It raises a usability problem since individuals have to remember different passwords for various online services every day. For example, some governmental services require smart card-based e-signatures, such as e-prescriptions services used by doctors in the health sector. Simultaneously, people also need to have national ID cards to identify themselves officially, which is also capable of e-signatures. In addition to that, to handle their financial transactions, Turkish users have different passwords for each legal bank account they own because their bank may not provide an electronic signature login option. As a result, people are obliged to manage all these different passwords and identification documents in their daily lives.

Besides usability and fragmentation problems Turkish users face, the existence of different e-signature technologies results in other technical structures (Zhang & Xiao, 2020; Wang et al., 2019) that needed to be combined to establish a Turkish eID environment capable of cross-border validation. This non-standard and separate big data not only affects the citizens and also brings forth vulnerability problems for organizations to think about, such as struggling to manage the sheer volume of vulnerabilities (Tang, Alazab & Luo, 2017; Dzikrullah & Rinjani, 2017). The main limitations in these services are technical operability, cross-border interoperability, usability, and lack of legal harmonization. In the meantime, in Turkey, Electronic Signature Law No. 5,070 imposed in 2004^20^ 20Electronic Signature Law No.5070, https://www.resmigazete.gov.tr/eskiler/2004/01/20040123.htm, retrieved 25.05.2021. is still in force. Besides the national ID card system, dongle-based methods, SIM card-based methods, and recently oAuth methods are still used to authenticate users in Turkey’s different platforms. On the other hand, despite the eIDAS Regulation effective from 2016, there has been no change in Turkey’s e-signature law yet to comply with the eIDAS Regulation.

After the preparations of an electronic signature, law No. 5,070 in Turkey; electronic signatures have started to be used since 2004. After that, developments in e-government applications have also accelerated. For example, Turkey’s e-government project, which aims to provide joint public services from a single point, became operational in 2008 with 22 services (Information Society Statistics, 2011). Since January 2017, applications have reached 3,027 services (ITU, 2016). Mobile electronic signature infrastructure was put into practice with telecommunication companies in 2007^21^ 21Regulation on the Procedures and Principles Regarding the Application of Electronic Signature Law, https://www.resmigazete.gov.tr/eskiler/2005/01/20050106-15.htm.. Today, three leading telecommunication companies serve mobile e-signatures in collaboration with six electronic signature certification authorities. According to the Turkish Electronic Communication Sector/Quarterly Market Data report of the Information and Communication Technologies Authority, the percentage of mobile signatures produced over electronic signatures in Turkey is only 14%, until the year 2019 (Quarterly Market Data Report, 2019). Turkey’s citizen card studies started in 2008 with the Scientific and Technological Research Council of Turkey (TUBITAK-UEKAE) (Mutlugün & Adalier, 2009). Citizen cards officially started to be distributed to citizens all over the country through the general directorate of population and citizenship affairs at the beginning of 2017. According to the public employees’ e-population union^22^ 22TC Icisleri Bakanligi-Nufus ve Vantandaslik Genel Mudurlugu-TC Kimlik Karti, https://www.nvi.gov.tr/tc-kimlik-karti., the number of distributed citizenship cards reached 37 million (almost half of the 82 million in Turkey) in 2019. Along with these solutions, Turkey also participated in some international projects initiated among European countries. Although different organizations from the EU carry out these projects, their joint mission was developing typical specifications for secure and mutual recognition of national electronic identities between countries. Other sectors adopting various methods increase the available system’s complexity in implementing other e-signature solutions. Besides that, these new technologies’ requirements can ensue as software or hardware requirements, which hardens eID providers’ and individual’s adaption processes. The need for a simple and inclusive identity authentication method arises day by day under these circumstances in Turkey. Therefore, providing architecture with higher usability and possibly an international solution for identity authentication is aimed at this study.

## A Server-Based Electronic Identification and Signature Scheme

A design of a server-based signature scheme should consider two essential steps to protect signature creation data; a robust authentication mechanism and the protection of the authentication data during communication between the user and the interface for server signature creation device (Orthacker, Centner & Kittl, 2010). In different schemes, the signature activation mechanisms and the approaches to ensure the sole control requirement vary greatly (Leitold & Konrad, 2019). In our scheme, as in the Australian scheme, a mobile phone (without any cryptographic module) is required as possession of the user. Only an OTP connection is sufficient instead of employing hardware secure elements. In the following subsections, a scenario to integrate remote signing strategy into the existing system in Turkey has been given; firstly, requirements are listed, then the four phases of the identity life-cycle are examined.

Recently, server-based approaches are becoming more common across countries. The difference is mainly on the level of the identity proofing and verification phase. In the proposed system, the server-based method relies on the concept that cryptographic operations are handled in hardware security modules (HSM) instead of users’ local devices such as SIM cards or smart cards. Mobile phone identity proofing is done; however, the smartphone is not necessary; only OTPs are used. With the server-based signing, signing keys are held on a service provider’s HSM (Orthacker, Centner & Kittl, 2010). With this approach, the need for users to handle their own private keys is eliminated. Austria is one of the exemplary countries where the server-based eID concept is implemented and used for several years countrywide (Orthacker, Centner & Kittl, 2010; Stranacher et al., 2013). They have made the necessary legal arrangements in its electronic signature law allowing the usage of server-based methods in the country in addition to the leading national ID card solution (Orthacker, Centner & Kittl, 2010). In Turkey, there has been no attempt to implement any national server-based eID structure so far. A server-based eID approach is adopted for interoperability and also to increase usability. Despite differences, the modular and flexible solution of Rath et al. (2014) eases the implementation of the method and helps us establish a preliminary eID infrastructure for Turkey.

**Figure 1:**
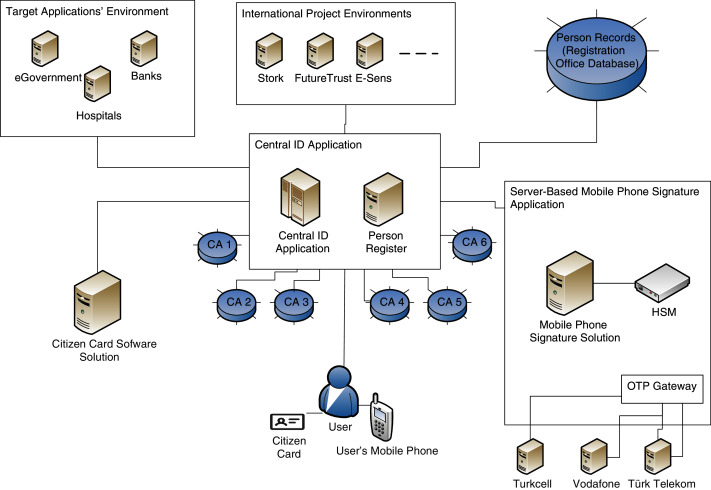
eID infrastructure.

In this part, requirement analysis is conducted, and primary necessities of the Turkish eID scheme are specified as below;

- A central ID application in eID structure that provides an integration of Citizen Card Software’s systems, General Directorate of Population and Citizenship Affairs’s system, and e-signature data kept on certification authorities (CA) is given as in Fig. 1.
  –It should support both citizen card signatures and server-based signatures.
  –The application should manage a common infrastructure between certification authorities, General Directorate of Population and Citizenship Affairs securely.
  –It should include a person register component, and it should support the registration types; face-to-face registration, self-registration (using existing qualified eIDs), and registration *via* trusted organizations (such as a bank or a university).
  –Person register application should serve as a common point of contact enabling all outside ID sources to connect with it. It should provide a standardized ID infrastructure.

- A server-based signature application that is capable of creating server-based e-signatures in a HSM device is necessary, as in Fig. 1.
  –HSM usage in the server-based signature solution is needed to improve the security level.
  –Web interfaces (usage) are necessary to render the server-based signature usage.

### Enrollment phase

The enrollment phase is the first phase of an eID scheme; the user has two options to register; use an existing eID or apply for a new eID (providing his/her legal identification document).

- **Application *via* new eID and Registration:** The user applies registration authority with his/her breeder document such as an official ID, a passport, or a driving license. The registration officer (National Identity Provider) conducts face-to-face validation/verification of the user. Officer manually registers the user in the person register system with the user’s specific data (*e.g*., name, national identification number, phone number, etc.) *via* a web-based form. A one-time code is sent to the user’s provided mobile phone number for validation. If the user’s one-time code is valid, a unique identification record and an activation code have been created in the person register application.
- **Registration *via* Existing eID: **If the user wants to use his/her existing eID information stored in his/her national ID card, firstly, he/she connects his/her ID card to a card reader. Then he opens an online person register application provided by the central ID application component, an online environment verifying the user’s identity and enabling users to complete the registration on their own. The user creates a unique identification record in the person register application *via* a web-based form. The user’s existing eID is validated through this web service and the user’s certification authority. If the existing eID is valid, a one-time code is sent to the user’s provided mobile phone number for validation. If the user’s one-time code is valid, a unique identification record and an activation code have been created in the person register application.

Created identification record and activation code are shared with the server-based mobile phone signature application so that the user can log in to the proposed server-based mobile phone signature application with his/her phone number and activation code. Also, an activation code is sent to the user’s mobile phone.

### Identity proofing and verification phase

After a successful login to the server-based e-signature application, the user can start the signature creation process. A unique identification record is already shared with the server-based e-signature application, and there is no eID or signature created up to this point. The user fills an activation form and determines a unique secret password (with a revocation PWD) to create an eID for himself/herself with the help of a CA. If the user already has an existing eID (Registration Alternative 2), he/she does not need to choose a certification authority. If the user does not have a current eID (Registration Alternative 1), he/she selects a certification authority in the activation form. The user is directed to the chosen certification authority’s payment system to complete the necessary payments. In the current situation, CAs in Turkey produce certificate and revocation status records and sign them with their own signature creation data. They are also responsible for the distribution, renewal, and disclosure of revoked certificates to all parties. In our proposed scheme, they are responsible for the same tasks, but they don’t give the users’ key pairs in a dongle or smart card; they share the information with the central eID application.

After the activation form is completed, a one-time password (OTP) is sent to the user’s provided mobile phone number for validation. If the user’s one-time code is correct, a signing key-pair generated in the HSM and an encryption key pair for the user are created in the server-based application’s server with CA interaction. Note that, in this scenario, encryption and signing key pair are not the same. After an eID is created for the user, it contains a specific phone number and secret password information (plus revocation password) besides the user’s personal information transferred from the person register. At the end of the process, created eID and keys are encrypted; the user’s private signing key is stored in the HSM Module (encrypted with HSM’s master key). The user’s private key is encrypted using symmetric encryption with the hash of the user’s secret password. All encrypted entities are stored under the server-based application securely.

The encryption keys are stored encrypted in the application server using the password of the user and the signatory’s mobile phone number. A secret password has been chosen by the user while creating eID and it is stored in the system by hashing. By the way, HSM can only decrypt if the user is authenticated. Therefore sole control is on the user. The HSM initiates a qualified electronic signature by creating a challenge and a verification code, both to be delivered to the signatory’s mobile phone.

### Issuance (credential management)

In this phase, creating and distributing credentials are regulated; however, there are no physical credentials to develop/distribute in such a system. It uses the credentials already distributed. The maintenance of HSMs is central. The revocation should be legally regulated, CAs should publish revoked certificates.

### Identity authentication

If a user wants to sign a document using an electronic signature, he/she may sign with server-based or national card-based authentication. If server-based signing is chosen by the user, in the web interface of the server-based e-signature application, the user is asked to provide his/her phone number and the secret password. Phone numbers and secret passwords are sent to the server-based e-signature application server *via* a secure connection. If the given information corresponds to an eID record in the system, the user receives an OTP to his/her registered mobile phone. After that, the user is required to provide the correct OTP in the web interface. During this process, a unique reference value is also created. This value is shown to the user in both the web interface and in the SMS message. This reference value is used to prevent man-in-the-middle attacks and to ensure perfect forward secrecy^23^ 23Guidance for the application of the levels of assurance which support the eIDAS Regulation, https://ec.europa.eu > download > attachments.. After all of the provided information is verified within the system. Then, the electronic signature is performed on behalf of the user inside the HSM module. Then signed data is transferred to the target application through the central ID application. After this phase, authorization and identity management is the ongoing process of retrieving, updating, and deleting identity attributes and policies governing users’ access to information and services.

## Discussion

### Technical applicability

The following technical factors should be considered to evaluate the method’s availability in Turkey. Since 2017, Turkey has distributed about 37 Million Citizen cards with e-signature functionality to its citizens (General Directorate of Population and Citizenship Affairs of Turkey, 2019). The proposed solution combines the citizen card concept and the server-based mobile phone signature concept. Therefore, it is technically not difficult to implement this method as there is no need to change the existing infrastructure.

According to the FORMIT (2013) foundation’s study report prepared by the European Commission in 2013, in the matter of e-signature products, three factors; “usability”, “security”, and “interoperability” need to be taken into account and harmonized in order to find the optimal point of balance. Therefore, the proposed system’s main contributions for Turkey’s electronic signature scheme are usability increase and cross-border interoperability (model is applicable to Pan European Proxy Service (PEPS) model (Lenz & Zwattendorfer, 2016)), besides a sufficient level of security increase accordingly.

#### Usability

A centralized e-identity management technology enabling single eID for multiple services may free people from the burden of creating different accounts and managing multiple passwords for services (Ruica, Pura & Aciobanitei, 2020). Since no reader or additional software is required of users, a server-based signature is a comparatively cheap, user-friendly, and flexible solution. The mobile devices do not need special-purpose hardware (such as smart-card readers or Trusted Platform Module chips) and special-purpose SIM cards. The proposed solution can be implemented with non-smartphones as well. This convenience improves usability by extending the sphere of influence.

#### Cross-Border Functionality

The solution provides self-registration functionality where identification attributes can be read directly from the foreign ID card with a qualified certificate, allowing cross-border authentication (Stranacher et al., 2013; Rath et al., 2014). Central ID management prevents different levels of information in different systems at a national level. It supports the collection of homogeneous data for the Member States at the European level. With the introduction of eIDAS Regulation replacing the old eSignature Directive 1999/93/EC (https://eur-lex.europa.eu/legal-content/EN/TXT/?uri=celex\%3A31999L0093, retrieved 24.05.2021), across Europe, adopting the server-based eID methodology provides a legal structure for Turkey’s e-signatures, and international eID schemes would be described and supported.

#### Security

According to Sullivan (2018), the digital identity concept is supported not only for cost and efficiency purposes but also to reduce fraud. Different eID solutions are applied by various sectors such as banks, trading sites (shares, stocks), ticket sales (hotels, airplanes), shopping sites, the health sector, and government institutions in Turkey. This situation causes different security levels for other applications. A hardware security module (HSM) is a physical computing device that contains one or more secure cryptoprocessor chips. The server-based concept relies on HSM modules instead of SIM cards or smart cards to guarantee the security of the cryptographic operations (Orthacker, Centner & Kittl, 2010). With the remote signing, signing keys are held on a service provider’s HSM. Therefore the need for users to handle their own private signing keys is eliminated. On the other hand, typical attacks on possession-based authentication factors are theft, duplication, or tampering; however, a server-based system against such attacks is indisputable.

#### Accessibility

Besides used technology, technical background for exceptional cases is also an essential aspect of the eID issue. In the event of losing or forgetting the actual secret password, the user can demand to change his/her password through a revocation password in the presented solution. Suppose the revocation password is forgotten or lost too. In that case, the person must pay a visit to the registration authority with an identification document to authenticate face to face.

In Turkey, disabled people are provided with their identification documents, but they may not perform legal processes due to their limitations. In that case, their legal guardians perform their necessary operations on behalf of them. Disabled people can still register the application and create a unique identification record for themselves, but they can not apply e-signatures. If a signature is needed for application, their legal guardian’s signature is necessary. Today in Turkey, underage people (age of eighteen) have their identification documents for themselves; however, they can not legally provide e-signatures.

### Legal applicability

Although the old Directive (https://eur-lex.europa.eu/legal-content/EN/TXT/?uri=celex\%3A31999L0093, retrieved 24.05.2021), does not comply with remote electronic signatures, the eIDAS Regulation (https://ec.europa.eu/futurium/en/content/eidas-regulationregulation-eu-ndeg9102014), also endorses this. eIDAS aims to reduce bureaucracy, make processes less costly, and make individuals’ and companies’ lives easier. For these purposes, eIDAS gives countenance to the use of server-based e-signing services to manage private keys on behalf of the users (eIDAS, 2009; Polanski, 2015). Since there is already a need for a structure compatible with eIDAS, the proposed design is a good solution to handling legal and technical obstacles. The fact that Austria’s technical infrastructure (server-based e-signatures using HSM) is now considered a reliable and valid system according to eIDAS (2009) supports the applicability of the proposed solution for Turkey (Polanski (2015)).

As of the date of its entry into force, the eIDAS Regulation revokes the EU Directive 1999/93/EC (https://eur-lex.europa.eu/legal-content/EN/TXT/?uri=celex%3A31999L0093, retrieved 24.05.2021), which is the basis of the legal and technical framework of electronic signatures in Turkey. In this context, the “Report on the Regulation of Trust Services” was prepared by the Information Technologies Department of Turkey in 2018 (Activity Report 2018, 2018). Within the scope of harmonizing Turkey’s laws with EU legislation, updates on trust services in Turkey’s laws in line with eIDAS must be completed.

Besides these, the proposed server-based eID infrastructure is a product of a public-private partnership in Austria (EGIZ, 2009). Polanski’s study mentioned that, the unification of e-trust services requires adoption and cooperation between states and private parties rather than complicated implementations (Polanski, 2015). Public-private partnership projects should be initiated to combine public and private sectors and create a homogeneous structure throughout the country. These projects should be managed and audited by the government. Governmental management of such projects is essential. However, the legal framework is constituted in a timely manner, the use of e-signature may not reach the expected levels due to insecurity in new technologies (Kabasakal, 2007).

## Conclusions

In this study, we examined previous research in the literature on electronic identity (eID) credentials technologies and the projects carried out in Europe. We focused, especially on server-based e-signing methods. In the light of these reviews, we examined the applicability of a server-based mobile electronic signature model without disrupting local initiatives as a case study on the example of Turkey. Our analysis found that although Turkish citizens’ identity cards have an e-signature infrastructure, this feature is not yet widely used. In Turkey’s current eID structure, it is seen that different types of electronic signature solutions are tried to be implemented in various sectors and applications. However, none of them seem to have spread as planned. To address the adoption (Marsalek et al., 2017) problem in Turkey, we made a detailed analysis of the Austrian server-based eID approach, a combination of national identity cards and mobile signatures. As eIDAS allows the use of remote signing services that manage keys on behalf of service users, we have identified the primary requirements to evolve the eID infrastructure of the current Turkish eID plan towards a server-based approach. It has been observed that a server-based eID scheme can be achieved by making some changes in the existing Turkish eID structure, taking into account the existing structure and needs. It was concluded that the proposed eID structure is applicable to Turkey’s legal and technical frameworks. The proposed centralized eID architecture does not provide a more secure environment than existing QES solutions (Theuermann, Tauber & Lenz, 2019), but all industries/applications (private or public) can benefit from a higher user experience. In addition, server-based solutions are based on a centrally implemented SSCD that is shared among all users. Storing and processing security-critical data in a secure central environment will also facilitate the management of the system. In conclusion, the proposed e-signature approach offers practical solutions to problems in usability and cross-border interoperability. Mobile devices, which are the most critical components of the server-based e-signature system, are widely used in societies today. With this high prevalence, using a server-based electronic signature with a mobile/electronic card may be the e-signature technology of the future. Government and private sector actors must work together to achieve a server-based e-signature system. Integration should be established between the country’s central registration authority and certificate authorities, and legal regulations should be brought in line with international standards. During the study, the Austrian system and its applicability to Turkey were investigated. A significant difference between population sizes in this process (approximately nine times the population of Austria) revealed a possible technical performance limitation problem. It was concluded that more advanced and additional distributed systems could be considered to offer a solution to this problem (the distributed HSM design could be adapted to overcome the performance bottleneck). In summary, this study revealed that a server-based eID approach would contribute to developing a more user-friendly and flexible solution, but significant efforts still must be made to drive broad adoption.

## Additional Information and Declarations

### Data Availability

The following information was supplied regarding data availability: This is a literature review.
